# Supplementary material for: Large self-assembled clathrin lattices spontaneously disassemble without sufficient adaptor proteins
Source: PLoS Comput Biol. 2022 Mar 21;18(3):e1009969. doi: 10.1371/journal.pcbi.1009969 (PMC8979592; doi:10.1371/journal.pcbi.1009969)
Supplement: S1 Text — Text A: Supplemental Methods. Table A: Calculations of average energies per trimers assembled into lattices. Fig A: Numerical confirmation of expected kinetics. Fig B: Evaluating how association events that are rejected for producing significant orientational displacements affect reaction kinetics. Fig C: Kinetics of clathrin accumulation on membranes with changing model parameters. Fig D: Correlations between observed lag-time and steepness of initial growth from simulations vs predicted values from the simplified models of Eq 2 and Eq 3 of main text. Fig E: Growth mechanisms of clathrin lattices at the more “physiologic-like” geometries. Fig F: Growth of lattices with changing clathrin concentration, at fixed 1μM of adaptors. Fig G: Fraction of assembled clathrin in solution vs adaptor concentration. Fig H: Maximal lattices formed as AP2 concentration is varied, comparing two values of cooperativity in clathrin-clathrin contacts, ΔGcoop. Fig I: Reduced lipid populations or slower lipid binding rates slow the lag. (PDF) [file pcbi.1009969.s001.pdf]

Supporting Information for:

Large self-assembled clathrin lattices spontaneously disassemble without sufficient adaptor proteins

Si-Kao Guo, Alexander J. Sodt, Margaret E. Johnson*

* margaret.johnson@jhu.edu

This PDF file includes:

Text A: Supplemental Methods.
Table A: Calculations of average energies per trimers assembled into lattices.
Fig A: Numerical confirmation of expected kinetics.
Fig B: Evaluating how association events that are rejected for producing significant orientational displacements affect reaction kinetics.
Fig C: Kinetics of clathrin accumulation on membranes with changing model parameters.
Fig D: Correlations between observed lag-time and steepness of initial growth from simulations vs predicted values from the simplified models of Eq 2 and Eq 3 of main text.
Fig E: Growth mechanisms of clathrin lattices at the more “physiologic-like” geometries.
Fig F: Growth of lattices with changing clathrin concentration, at fixed 1μM of adaptors.
Fig G: Fraction of assembled clathrin in solution vs adaptor concentration.
Fig H: Maximal lattices formed as AP2 concentration is varied, comparing two values of cooperativity in clathrin-clathrin contacts, ΔG_{coop}.
Fig I: Reduced lipid populations or slower lipid binding rates slow the lag.
SI References

Other supplementary materials for this manuscript include the following:

S1 Movie. Video of the clathrin assembly matching the *in vitro* simulations, as shown in Fig 3 of main text.
(MP4)
S2 Movie. Videos of clathrin assembly at physiologic-like conditions, as a function of varying adaptor concentration (Fig 4 of main text).
(MP4)
S1 Table. Excel sheet containing the parameters for all simulations with discernable lag times and growth rates. Includes the corresponding observed lag times and steepness of initial growth from simulations, and predicted lag times and steepness from our theoretical expressions.
(XLSX)

Supporting Information Text

Text A Supplemental Methods

Maintaining a constant (fluctuating) clathrin concentration

The constant clathrin concentration is maintained by a zeroth order reaction producing clathrin at $k_{\mathrm{create}}=[\mathrm{Cla}]_{\mathrm{tot}}D_{\mathrm{Cla}}/z^2$, which approximates the timescales of diffusive flux across volume elements of height z. Simultaneously, clathrin is degraded in solution at a rate of $k_{\mathrm{destroy}}=D_{\mathrm{Cla}}/z^2$, where D_{Cla} is set to $13\mu m^2/s$. Clathrin on the membrane is not affected by the degradation reaction.

System parameters of the NERDSS simulation for assembly reactions.

Association events within a rigid complex are necessary to close polygons (like hexagons and pentagons). The reaction rates for these unimolecular reactions are derived based on the bimolecular rates, while allowing for the addition of positive or negative cooperativity via the parameter $\Delta G_{\mathrm{strain}}$ (see Ref [1]). These unimolecular reactions are attempted when reactive partner sites are within a maximum distance defined by the binding radius σ multiplied by a factor, bindRadSameCom [1]. The bindRadSameCom was set to 1.1 in our simulations of both the flat and the curved clathrin lattices. For the flat lattices which are free of defects, all hexagons can be closed by these reactions. For the curved lattices, larger defects may not result in the closed polygon bond forming due to the interfaces being too far apart.

NERDSS can reject associate events for two reasons:

1) if an association event causes components of the two complexes to sterically overlap, the event is rejected, and the complexes remain separate. NERDSS evaluates steric overlap after association by measuring distances between all centers of mass (COMs) in the new complex and defining a minimum distance, overlapSepLimit, that will determine the steric overlap[1]. The overlapSepLimit was set to 7nm in our simulations. The flat lattice simulations are not sensitive to this value, because the lattice is free of defects—overlapping clathrin always have a separation of 0nm and are thus always rejected. For the curved clathrin cages in solution, the lattice can have defects and we found that this size prevented two monomers within a cage occupying the same space[1].

2) if an association event causes elements of either complex to undergo large-scale displacement as they snap into the proper orientation, the move is rejected. This effectively only applies to large complexes, where sites distant from the COM can potentially displace significantly upon association. This is enforced by a cutoff value, where events that result in shifts of an interface on either complex by scaleMaxDisplace* <RMSD> are rejected, because the displacements are not physical. <RMSD> is calculated from $(6*D_{\mathrm{eff}}*\Delta t)^{0.5}$ in 3D, and $(4*D_{\mathrm{eff}}*\Delta t)^{0.5}$ in 2D, where D_{eff} is the effective translational diffusion coefficient of one complex [2], accounting for rotational diffusion [1]. The scaleMaxDisplace was set to 10 in our simulations (see Fig B in S1 Text).

Mesh resolution in the continuum membrane model

Mesh resolutions were tested from 448 to 1344 triangles (ca. 1093-364 nm^2 per triangle) with variation in the energy with mesh resolution on the order of 5% for highly deformed structures. The energy generally decreases with increased mesh resolution as it has more freedom to adapt to the cage. The energy is determined as a function of the mesh vertex coordinates that are input to the subdivision scheme.

During minimization, the nearest point on the membrane, tagged in local surface coordinates (face, and internal triangular coordinates) was saved during 50 "sub-iterations" of the Broyden-Fletcher-

Goldfarb-Shanno (BFGS) conjugate-gradient class algorithm. Following these sub-iterations, new nearest points were computed for each virtual site. In this way, efficient BFGS minimization could proceed without the somewhat expensive recalculation of the nearest sites.

Transition matrix and probability distribution of clusters with different size NERDSS tracks all complexes formed based on the number and type of components. We calculate the transition matrix T(Δt) using a small time interval for high resolution, Δt=150μs, to measure growth probabilities and lifetimes (Fig C in S1 Text). The diagonal of T(Δt), T(Δt)$_{n,n}$ contains the probability that one cluster stays the same size n in a time step Δt. Off the diagonal are transitions between clusters of all sizes, from n=1 to n=N_{max} (total number of clathrin) including monomer and non-monomer transitions. P(n) is the probabilities of observing a cluster with size n and can be calculated from summing T(Δt) over the column n, and then normalizing over all n.

Estimating n_1 and n_2 lattices sizes from probability distributions. To extract the lattice sizes where the barrier to initial growth flattened, we analyzed the $-\ln(P(n))$ distribution for each trajectory, where $P(n)$ is the normalized histogram that counts occurences of assemblies of size n. We chose a plateau value in $-\ln(P(n))$ for each trajectory, and where this value was first crossed determined n_1, and where it was last crossed (at the descent into the stable well) determined n_2. The plateau value was chosen based on where $-\ln(P(n))$ transitioned to noisy fluctuations, where we note that choosing a higher plateau value would predict slightly larger values of n_1 and smaller values of n_2. For the equilibrium distributions, $P_{\mathrm{eq}}(n)$, there was typically a gap where no lattices of intermediate sizes were observed. The start and end of this gap were used to determine n_1 and n_2. These values were then averaged over 4 trajectories, with standard errors of the mean (SEM) shown in the plot of $-\ln(P(n))$ and standard deviation (SD) shown in the plot of n_1 and n_2.

Phenomenological fit to lag and initial growth rate

Lag time: The lag time was fit to a sum of timescales, each of the form of (*rate* x *concentration*)$^{-1}$. To describe the physiologic-like simulations, a third timescale was added to account for binding of the adaptor proteins to the lipids. This term was not needed to describe the *in vitro* data, as adaptors were already affixed to the surface (so this third timescale is zero).

$$\tau_{\mathrm{CLA-loc}} = \frac{c_1}{\rho_{\mathrm{AP}} k_{\mathrm{AP-CLA}} [\mathrm{CLA}]_{\mathrm{bulk}} A} \qquad \text{(A1)}$$

$$\tau_{\mathrm{nuc}} = \frac{1}{f_{\mathrm{coop}} k_{\mathrm{CLA-CLA}} [\mathrm{CLA}]_{\mathrm{bulk}} \mathrm{DF}^{1/\delta} \exp\left(-\frac{\Delta G_{\mathrm{strain}}}{a k_B T}\right)} \qquad \text{(A2)}$$

$$\tau_{\mathrm{AP-loc}} = \frac{c_3}{[\mathrm{PIP2}] k_{\mathrm{AP-PIP2}}} \qquad \text{(A3)}$$

$$\tau_{\mathrm{lag}} = \tau_{\mathrm{CLA-loc}} + \tau_{\mathrm{nuc}} + \tau_{\mathrm{AP-loc}} \qquad \text{(A4)}$$

The dimensionless fit parameters ($c_1, \delta, a,$ and c_3) were found using nonlinear fitting in MATLAB. First, we collected the $[\mathrm{CLA}]_{\mathrm{mem}}(t)$ vs t from our reaction-diffusion simulations, and fit them to Eq 1 in the main text to extract the observed values of τ_{lag} and initial growth steepness, kE. For the physiologic like simulations at low adaptor concentration, the kinetics could not be fit well to this function, so we did not use those simulations in our global fit to Eq A4 in S1 Text. In total, 80 different simulation points were used in the global fitting (Table S2). Second, the global fitting to the parameters of Eq A4 in S1 Text was performed by

inputting the variables
$f_{\mathrm{coop}}, k_{\mathrm{CLA-CLA}}, [\mathrm{CLA}]_{\mathrm{bulk}}, \mathrm{DF}, \rho_{\mathrm{AP},}\ k_{\mathrm{AP-CLA}}, \Delta G_{\mathrm{strain}}, [\mathrm{PIP2}], k_{\mathrm{AP-PIP2}}$, as the known independent variables for each simulation, along with the observed values of τ_{lag}. The correlation between the observed lag times and the model predictions of the lag time using Eq A4 in S1 Text are in Fig C in S1 Text. Optimal fit values to $c_1, \delta,$ and a are printed in Eq 2 of the main text, and $c_3 = 0.4$.

Steepness/Initial growth rate: For steepness of growth, we used a mass-action type expression (proportional to rate x concentration). This function similarly had three terms for the full system, with the third term only needed when binding to lipids occurred for the same reason as above: in the *in vitro* simulations, the third term is zero.

$$kE = \frac{d[\mathrm{CLA}]_{\mathrm{mem}}}{dt} = k_{\mathrm{AP-CLA}}\rho_{\mathrm{AP}}[\mathrm{CLA}]_{\mathrm{bulk}}\mathrm{DF}^{\frac{1}{\eta}} + c_2 \mathrm{k}_{\mathrm{CLA-CLA}}[\mathrm{CLA}]_{\mathrm{bulk}}^2 f_{\mathrm{coop}}\mathrm{DF}^{\frac{1}{\beta}}\exp\left(\frac{\Delta G_{\mathrm{strain}}}{bk_BT}\right)V/A + c_4 k_{\mathrm{AP-PIP2}}[\mathrm{PIP2}]\rho_{\mathrm{AP}} \qquad \text{(B)}$$

Fit parameters were again found using nonlinear fitting in MATLAB against the same 80 different simulations points. Now we used the observed value of *kE* and fit to Eq B in S1 Text, with correlations shown in Fig C in S1 Text. This function fails to fully capture the dependence of the steepness of initial growth on cooperative clathrin-clathrin growth, systematically underpredicting growth rates with higher clathrin concentrations. Optimal fit values for $\eta, c_2, \beta,$ and b are printed in Eq. 3, and $c_4 = 0.00078$. The steepness is measured in copies/s/μm^2. We note that the dimensionless c_2 value is fit using concentrations in μM, *V* in units of μm^3, and *A* in units of μm^2. Cancellation between the units of μm^3 and μM is a factor of 602, which is included in c_2.

Energies per trimer in clathrin lattices

To calculate average energies per trimer in a lattice, we use approximate expressions derived assuming the clathrin grow into a compact lattice, maximizing formation of hexamers [2]. We thus use:

$$N_{\mathrm{bonds}} = 11 + 3.5(N_{\mathrm{hex}} - 2) \qquad \text{(C1)}$$

$$N_{\mathrm{hex}} = \frac{1}{2.5}(N_{\mathrm{trimer}} - 10) + 2 \qquad \text{(C2)}$$

Supplemental Table

Table A. Calculations of average energies per trimers assembled into lattices, due to bonds formed and hexamers formed. Counts from Eq C in S1 Text.

Counts per lattice			Energy per bond: -11.7kBT, Energy strain per hexamer: 6.9kBT		Energy per bond: -14kBT, Energy strain per hexamer: 4.6kBT		
N trimers	N hexamer	N bonds	Total energy on membrane (kBT)	Energy per trimer (kBT)	Total energy in solution (kBT)	Energy per trimer solution (kBT)	Total added stability per trimer (kBT)
5	0	4	-46.8	-9.36	-56	-11.2	-1.84
10	2	11	-114.9	-11.49	-144.8	-14.48	-2.99
20	6	25	-251.1	-12.555	-322.4	-16.12	-3.565
30	10	39	-387.3	-12.91	-500	-16.666667	-3.7566667
50	18	67	-659.7	-13.194	-855.2	-17.104	-3.91
100	38	137	-1340.7	-13.407	-1743.2	-17.432	-4.025
150	58	207	-2021.7	-13.478	-2631.2	-17.541333	-4.0633333
200	78	277	-2702.7	-13.5135	-3519.2	-17.596	-4.0825

Supplemental Figures

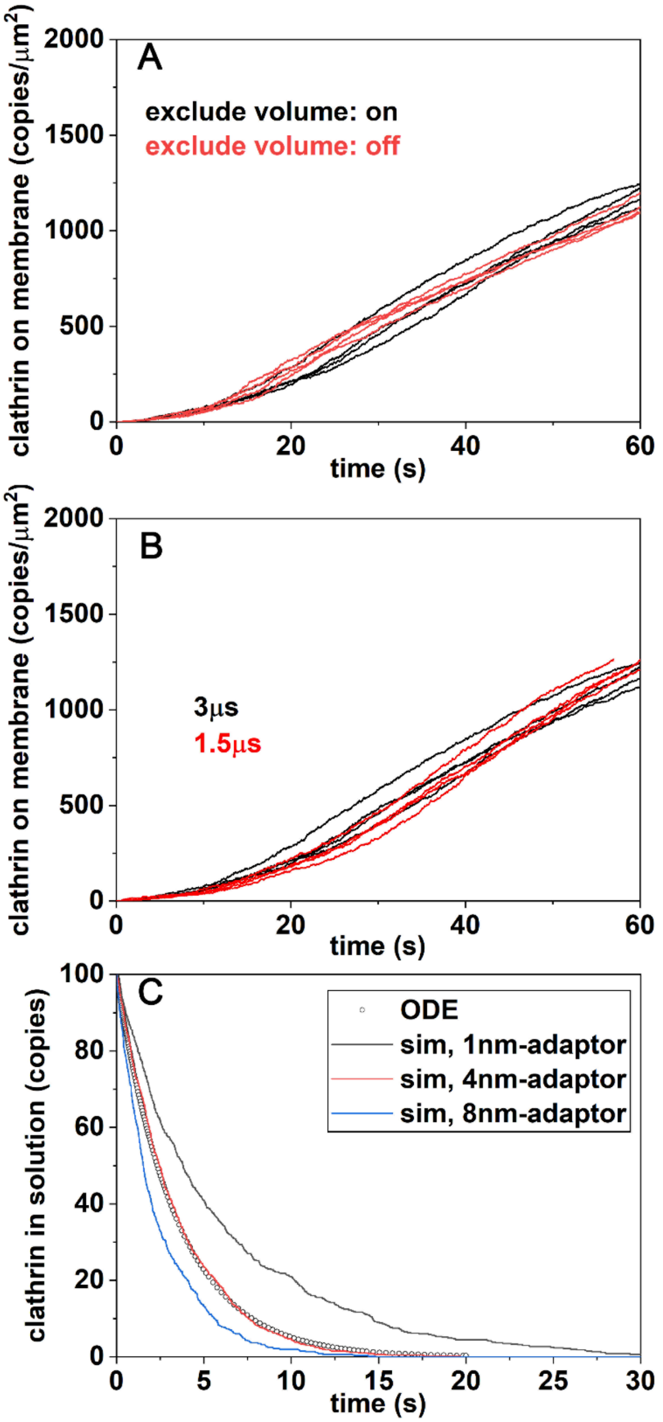

Fig A. Numerical confirmation of expected kinetics (A) Excluded volume between clathrin centers does not affect the kinetics. (B) The kinetics is independent of the simulation time-step. (C) The adaptor with a height of 4nm ensures accurate membrane binding kinetics as predicted given the corresponding macroscopic rate. This separation of the binding site above the reflective membrane surface is necessary due to the extended size of the rigid multi-site clathrin. The reflection of all the clathrin sites off of the membrane surface can reduce reactive flux between each reactive binding sites of clathrin that can bind

the membrane site. The system box is [1000, 1000, 1000] nm, including 100 copies of clathrin, 50000 copies of implicit adaptors on membranes, and the binary reaction between clathrin and adaptor. $k_{on,3D->2D}$=0.0012$\mu M^{-1}s^{-1}$. $k_{on,2D}$=6.63×$10^{-5}\mu m^2 s^{-1}$. k_{off}=0.03s^{-1}. The ODE is calculated using VCell[3] ignoring the spatial structure of molecules. The sim is 6 trajectories generated from simulation of NERDSS[1].

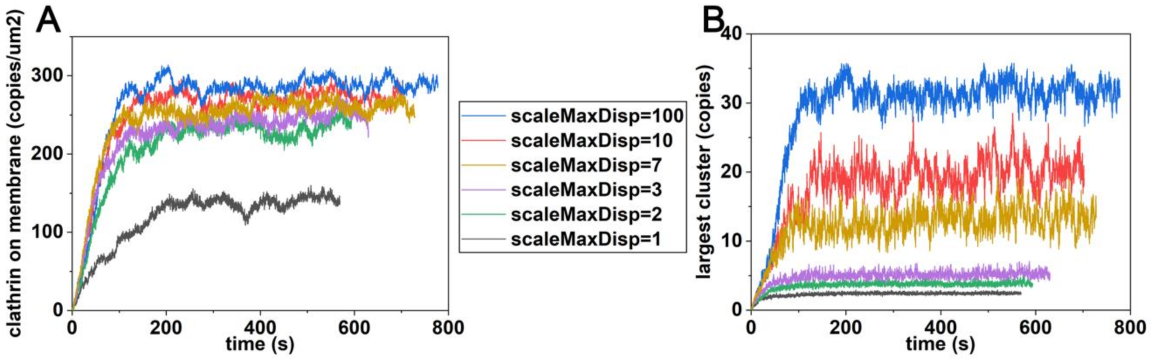

Fig B. Evaluating how association events that are rejected for producing significant orientational displacements affect reaction kinetics. (A) Our algorithm is designed to allow association events based only on separation, not orientation, and thus it is important for reproducing expected macroscopic kinetics that we do not reject too many moves based on orientational displacements. For scaleMaxDisplace=1, any association event that produces re-orientational displacements that are larger than the average value expected from diffusion during that time-step are rejected. This highly stringent constraint violates the assumptions of our model, and thus effectively reduces the rate of binding. With scaleMaxDisplace=10, we accept moves that are 10x the average displacement. We use a value of 10 because it produces similar kinetics to even larger values (including with no rejection at all). (B) Although this rejection criteria has minimal impact on the kinetics, it does influence the size of clathrin clusters that form, preventing two large structures, for example, annealing into one. The system box is [350, 350, 1000] nm, including 0.65μM clathrin and 1μM adaptor.

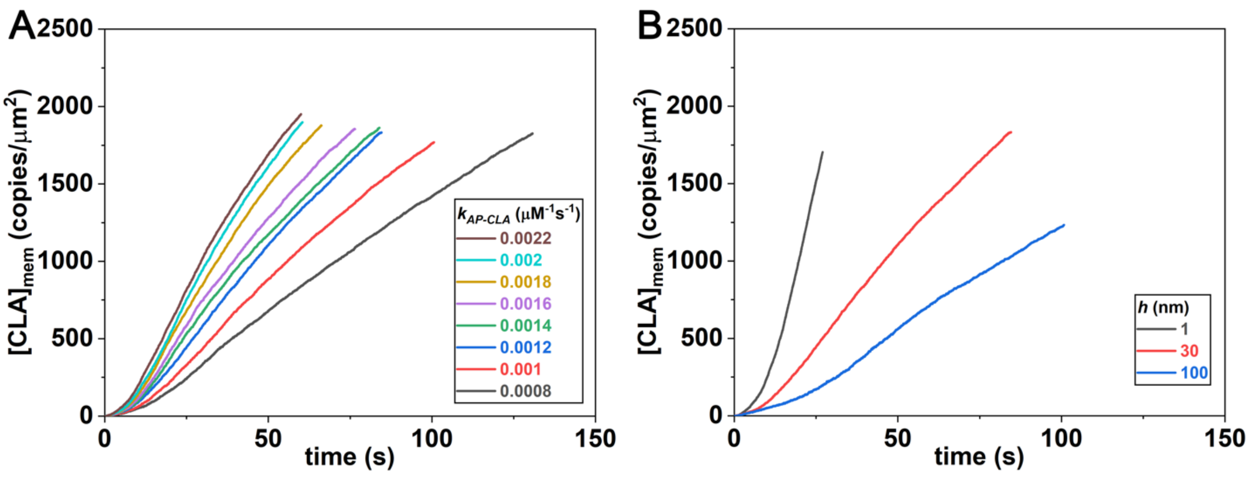

Fig C. Kinetics of clathrin accumulation on membranes with changing model parameters. (A) As the rate of binding between the adaptor protein and clathrin is increased, $k_{AP\text{-}CLA}$, the lag-time is shortening, and the growth steepness is increasing. (B) As the lengthscale *h,* which controls the 3D vs 2D K_D and on-rate, is shortened, the growth rate and steepness of growth increase substantially.

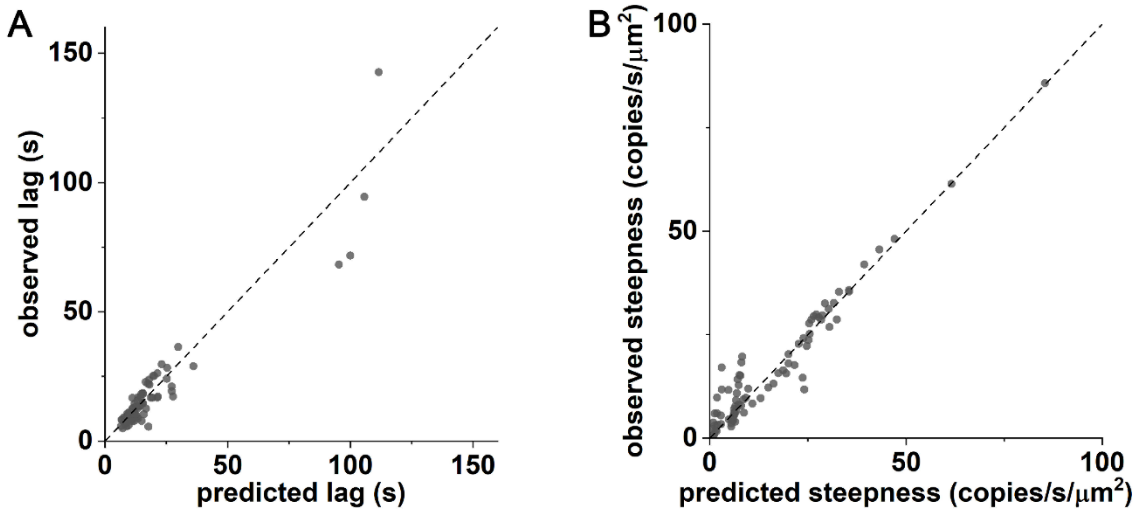

Fig D. Correlations between observed lag-time and steepness of initial growth from simulations vs predicted values from the simplified models of Eq 2 and Eq 3 of main text. (A) Predicted lag-time agrees quite well with observed lag, with results shown for 80 simulations (S1 Table). (B) Predicted steepness agrees relatively well with observed values. Same 80 simulations.

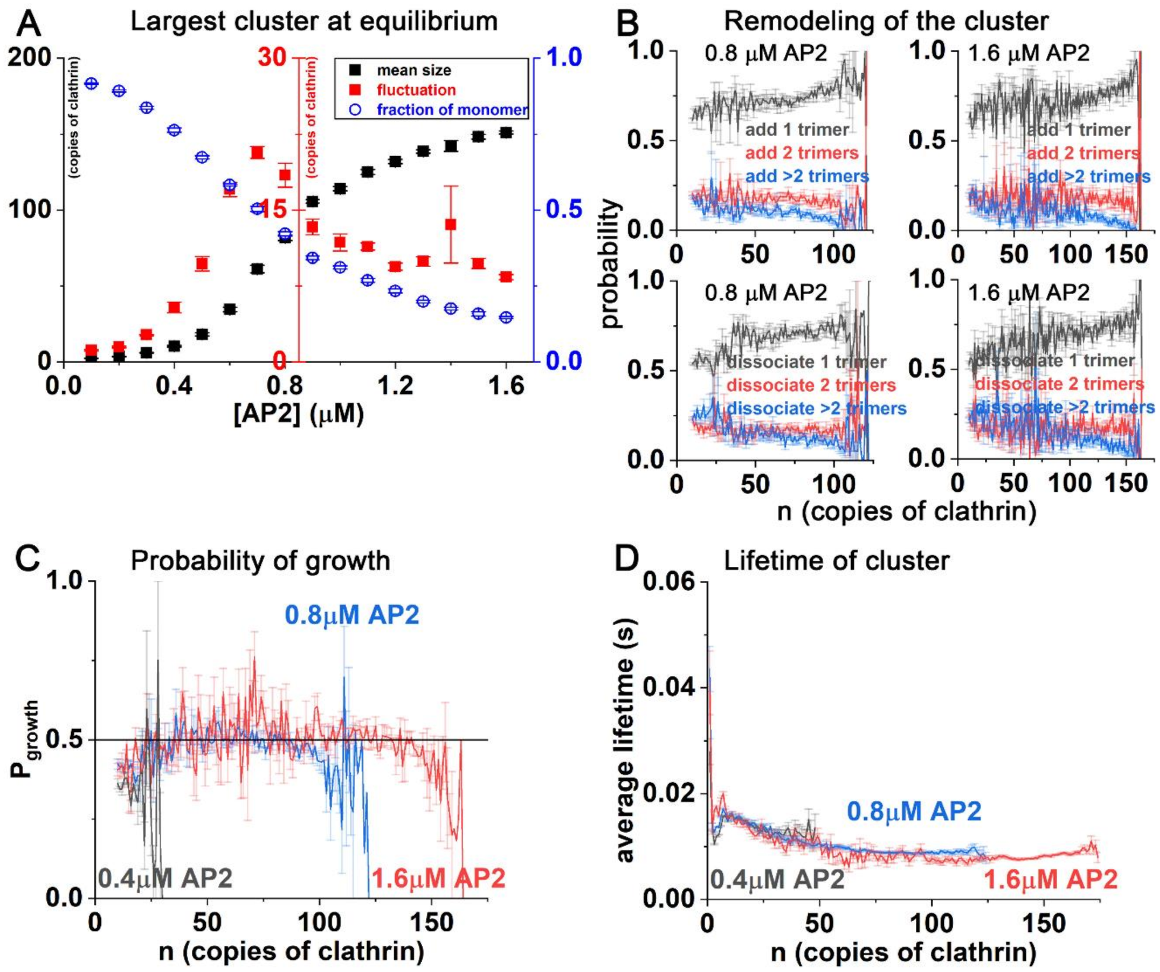

Fig E. Growth mechanisms of clathrin lattices at the more "physiologic-like" geometries. (A) Sizes of clathrin structures vs adaptor concentration. All data points have the same clathrin concentration (0.65μM). The mean lattice size increases with adaptors (black), with a corresponding drop in the fraction of monomers (blue). The fluctuations in the lattice sizes at equilibrium peaks at 0.7μM (red) indicating a transition point separating small and large stable lattices. (B) The growth of lattices of size n (x-axis) typically proceeds by addition or removal of a monomer (black data). About 20% of the growth is due to addition or removal of dimers (red), and the remaining is due to higher-order structures (blue). (C) Given that a transition occurs from a lattice of size *n* (x-axis) to a new size, we plot the probability that the transition causes a growth in size. For small lattices, the growth probability is less than 0.5, meaning that disassembly is more probable. For intermediate sizes, there is a small bias towards growth, until very large lattices form. This supports the dynamic remodeling observed in the simulations, as at all sizes, there is still a significant probability of disassembly. (D) The lifetimes of lattices of size *n* is ~0.01-0.02s, with monomers being the longest lived.

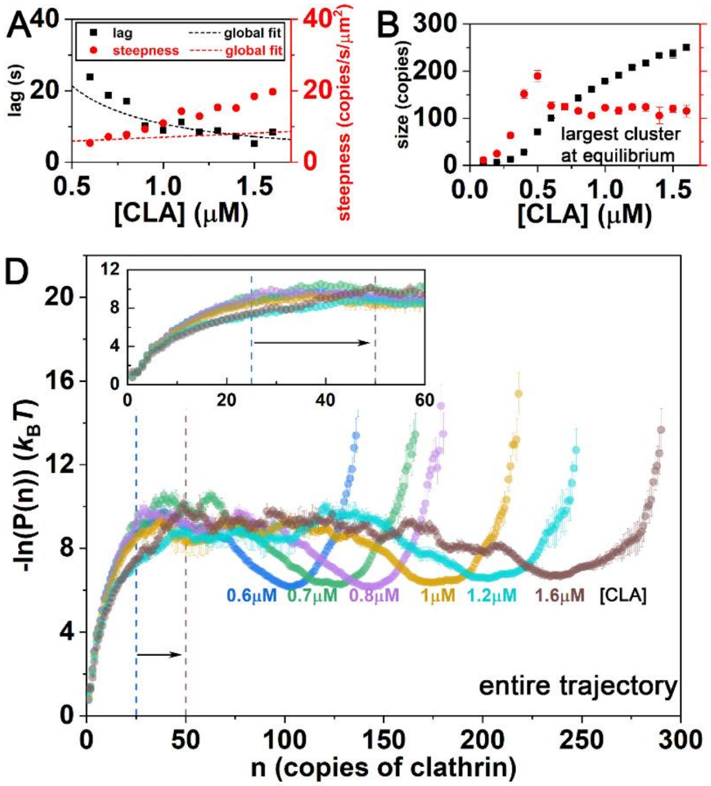

Fig F. Growth of lattices with changing clathrin concentration, at fixed 1μM of adaptors. (A) Lag time is well-described by the main text Eq 2/Eq A in S1 Text. Growth rate of Eq 3/Eq B in S1 Text does not as accurately capture speed-ups with higher clathrin concentration. The simple formula does not have sufficient cooperativity in clathrin-clathrin contacts in stimulating growth. (B) Lattice sizes increase with increasing clathrin concentration (black) with fluctuations peaking at 0.5μM clathrin. Prior to this point, maximal clathrin lattices are quite small (C) The fraction of monomeric clathrin decreases before flattening out (black). The stoichiometry of clathrin bound to adaptors keeps increasing, as more clathrin is added. It is not sufficient to have a stoichiometry of at least 1:1; at 0.2μM clathrin, the stoichiometry is more like 1:1.5, and yet no lattices form. Thus the total concentration is also a significant determinant of nucleation and growth. (D) The probability of observed cluster sizes *n* shifts to larger lattices with additional clathrin. The initial barrier also appears to shift to larger sizes with additional clathrin.

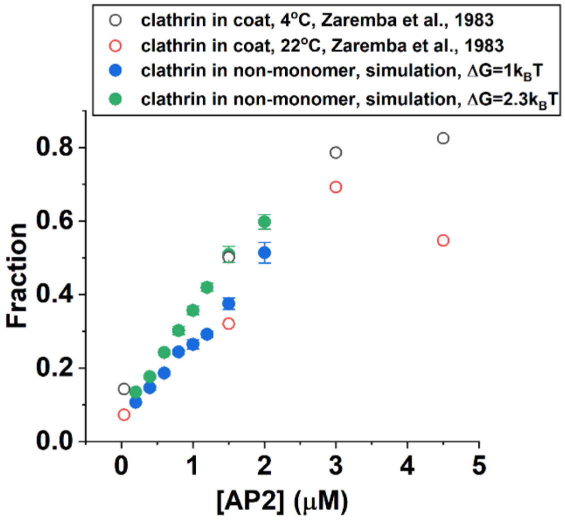

Fig G. Fraction of assembled clathrin in solution vs adaptor concentration. Experimental data is (open red and black circles) from Ref [4]. To convert from mg/ml of protein to μM, we used a molecular mass of ~650kDa for clathrin, and 50kDa for the adaptors, based on the values reported in Table 1 of Ref [4]. For the simulations, the clathrin concentration is 0.65μM. The clathrin concentration in our simulations is thus higher than in the experiments (~0.3μM), meaning that our model does not cause as strong assembly as observed experimentally. We note that in the experiments, the adaptors represented a mixture, that easily could have clustered amongst themselves, helping to nucleate lattices via cross-linking. Thus, we overall do not consider quantitative reproduction of these older experiments as critical as the recent fluorescence experiments.

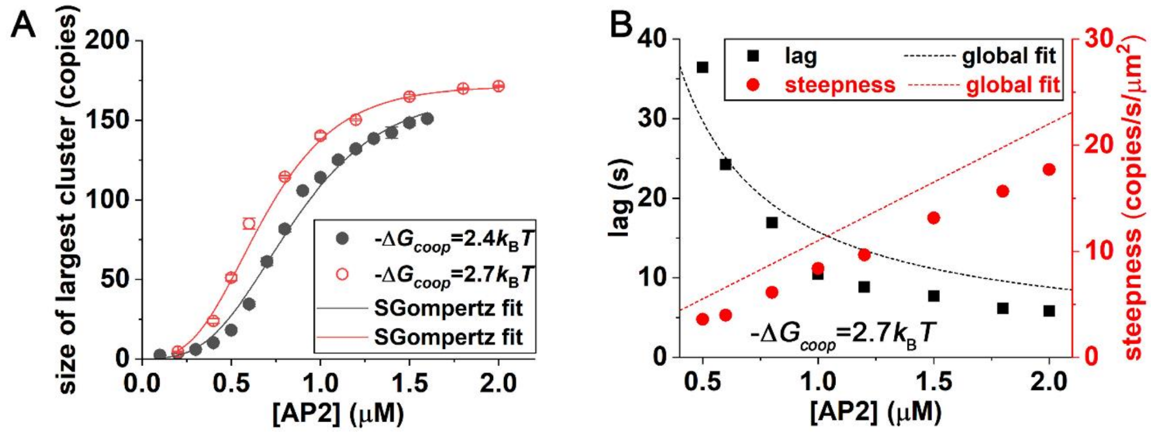

Fig H. Maximal lattices formed as AP2 concentration is varied, comparing two values of cooperativity in clathrin-clathrin contacts, ΔG_{coop}. (A) For adaptors that provoke stronger cooperativity in clathrin-clathrin assembly (red), the overall trend is the same, but the transition to larger and more stable lattices occurs at lower adaptor concentration. (B) For the more cooperative model, the lag-time and initial growth rate are described well by Eqs 2 and 3 of main text.

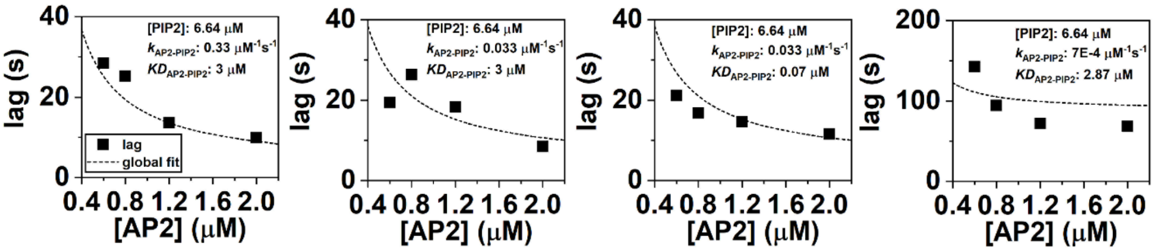

Fig I. Reduced lipid populations or slower lipid binding rates slow the lag. The lag-time are described well by Eq A in S1 Text as the lipid concentration, or rate and strength of adaptor-lipid binding are varied.

SI References

1. Varga M, Fu Y, Loggia S, Yogurtcu ON, Johnson ME. NERDSS: a nonequilibrium simulator for multibody self-assembly at the cellular scale. Biophysical Journal. 2020;118(12):P3026-40.
2. Johnson ME. Modeling the Self-Assembly of Protein Complexes through a Rigid-Body Rotational Reaction-Diffusion Algorithm. J Phys Chem B. 2018;122(49):11771-83.
3. Loew LM, Schaff JC. The Virtual Cell: a software environment for computational cell biology. Trends Biotechnol. 2001;19(10):401-6. doi: 10.1016/S0167-7799(01)01740-1. PubMed PMID: 11587765.
4. Zaremba S, Keen JH. Assembly polypeptides from coated vesicles mediate reassembly of unique clathrin coats. J Cell Biol. 1983;97(5 Pt 1):1339-47. PubMed PMID: 6138359; PubMed Central PMCID: PMCPMC2112702.
