# Supplementary material for: Large self-assembled clathrin lattices spontaneously disassemble without sufficient adaptor proteins
Source: PLoS Comput Biol. 2022 Mar 21;18(3):e1009969. doi: 10.1371/journal.pcbi.1009969 (PMC8979592; doi:10.1371/journal.pcbi.1009969)
Supplement: Attachment — Submitted filename: Response.pdf [file pcbi.1009969.s005.pdf]

Reply:

We thank the referees for the thorough reviews and have made significant revisions to our manuscript in response. To summarize, we added several new paragraphs to the Models and Methods section to provide more background, overview, and technical details about how the reaction-diffusion solver works, and how it specifically implements multi-component self-assembly. We added a figure to the supplemental (Fig S2) that shows how the kinetics vary with one of the assembly parameters, and we have fixed a few typos identified by the referees. Lastly, we have clarified several points made in the results based on the comments below.

Comments to the author:

Reviewer #1:

Guo, Sodt and Johnson present simulations of clathrin assembly at the cell membrane. Their simulations using NERDSS reproduce the experimental curves by Pucadyil and Holkar. While I agree with the authors that simulations provide a powerful tool to help understanding the experiments, I do not think this study is there yet.

General Reply: We thank the reviewer for taking the time to review our manuscript and raising these points. Please find our itemized responses in below and marked revisions highlighted in yellow in the manuscript.

Specific Reply to each comment:

Point 1:

Referee: *Ideally a study should be self-contained, which admittedly is difficult when using a complex model. The level of information provided in this manuscript is too low: it should, e.g., not be necessary for a reader to guess that the simulations are implemented as 1st order Brownian Dynamics.*

Reply: We realize that we relied significantly on referencing our previous work to explain the simulation method. We have now added more details about the implementation of

reaction-diffusion simulations, and the framework for the solver. We added two new paragraphs on pages 8-9 that explain how the reaction-diffusion solver propagates the systems through each time-step, and how orientation is dealt with between binding partners. We also added further sentences throughout the Model and Methods to additional points raised by the referees.

Point 2:

Referee: *The rotational diffusion coefficient of the triskelion, at 0.03 rad^2/sec, is a million times smaller than the value reported in the literature based on hydrodynamic calculations. Many triskelia will not even have rotated 180 degrees during the simulation of ~100 seconds.*

Reply: This was a typo--we thank the reviewer for pointing it out. We use units of $rad^2/\mu s$ in our simulation software. However, we reported the incorrect units in the manuscript, hence the appearance that it is exactly a million times too slow. We corrected the units in the manuscript to $rad^2/\mu s$. (Page 10)

Point 3:

Referee: *Experimental binding constants can only be plugged directly into the simulation when proteins are modeled as structureless particles. The directionality in the current model -- proteins only bind when they are positioned and oriented approximately correctly -- requires an adjusted reaction constant to reproduce the experimental binding constant.*

Reply: We see now that our description of the model was unclear in terms of how orientation is treated and how microscopic parameters of the model are related to macroscopic parameters measured experimentally. We have now clarified these points in the Models and Methods section in a new paragraph on page 9. Protein binding is dependent on the separation between the two reactive interfaces and is not constrained by their orientation. We describe the justification for this, which is in part due to the point made by the referee: with additional orientational constraints the relationship between

the experimental macroscopic rates and the microscopic parameters of the model were required to be adjusted.

Point 4:

Referee: *Binding of the triskelia to the membrane via AP2 alters the oriental distribution of these bound triskelia relative to the isotropic distribution in solution, and thereby affects their binding into aggregates. This change should not be introduced artificially with the tuning parameter h, as it is already automatically generated by the non-spherical protein model.*

Reply: With the reaction-diffusion model, the on-rates must be specified depending on the dimensionality of the search, they do not emerge naturally from sampling, and the same is true of the equilibrium constants (e.g., relating a model 2D to experimental 3D equilibrium constant). Once the molecules are restricted to the membrane, they perform a purely 2D search. In our algorithms, the reaction dynamics are evaluated based on solving a diffusion equation with reactive disks in 2D, and they require a rate constant with units of um^2/s. Thus, the 3D rate constant (units e.g. um^3/s) must be transformed by a lengthscale, *h*, ($k_a^{2D} = k_a^{3D}/h$) and similarly the 3D equilibrium constants. We assume the microscopic dissociation rates are the same in 3D and 2D, and there is no conversion necessary as they are 1st order reactions. Theory for converting from 3D to 2D equilibrium constants has been described for proteins at cell surface adhesions (Wu, Y. *et al*. Nature 475, 510–513 (2011)), where the lengthscale *h* was found to be on the nanometer/ molecular scale. Qualitatively, *h* is the height range in which the membrane-bound molecule is confined, which is not captured in an explicitly 2D reaction-diffusion model of binding when restricted to the surface. More detailed analysis is provided in (Weikl, T. R., Hu, J., Xu, G. K. & Lipowsky, R. Binding equilibrium and kinetics of membrane-anchored receptors and ligands in cell adhesion: Insights from computational model systems and theory. *Cell Adh Migr* **10**, 576-589 2016) and (Binding constants of membrane-anchored receptors and ligands: A general theory corroborated by Monte Carlo simulations. J Chem Phys 2015, Xu et al).

For 2D interactions involving clathrin, we tested values of *h*=1-100nm (Fig. $3D_3$ and Fig.S2B), the order-of-magnitude of the molecular clathrin length-scale.
We have now added a new paragraph on page 12 to clarify all of these points.

Regarding the orientational changes, On page 9 we have added new text explaining how the binding is dependent on the distance between binding interfaces, and not orientations. The influence of rotational diffusion on association events is accounted for following our previous derivation (Johnson, M, *J Phys Chem* **122**, 11771-11783 2018). The justification for this choice is twofold: it allows us to use analytical forms for reaction probabilities (this is important for our algorithm to take large time-steps while recovering exact association probabilities) and it allows us to directly predict how the macroscopic rates will depend on the microscopic parameters of the model.

For reactions that involve one molecule in 3D and one restricted to the surface, the search problem is driven by the 3D search, and uses the 3D constants. Due to the reflective surface of the membrane, there is a correction factor when extracting microscopic rates from the macroscopic rates, which was derived and validated in a previous work (Fu et al, *J Chem Phys* **151**, 124115 2019). Because clathrin has a large rigid structure, we validated in Fig S1 that its binding to the membrane recovered the proper kinetics expected from its specified rate constant (see text on page 14).

Point 5:

Referee: *What is the physical reality of the 'initial barrier to growth'? The free energy barrier to lattice formation is not lowered by the system being out of equilibrium.*

Reply: By 'initial' here we specifically meant as they transition from monomers to larger structures, so initial in terms of *n* not initial in terms of time. We have now added text to the Figure 5 legend to clarify on page 25-26. Out of equilibrium we use the analogous proxy measure -ln(P(n,t)), given that there is no definable free energy for a nonequilibrium system.

Point 6:

Referee: *In Eqs 2 and 3, why has the temperature (k_B T) become a fit parameter? Why do only two out of three terms acquire a fitted proportionality constant?*

Reply: We apologize, the missing k_BT in the exp() is actually a typo. We have corrected this in Eqs 2 and 3, and the supplemental material. The fit parameters are therefore the numbers that scale $\frac{\Delta G_{\mathrm{strain}}}{k_{\mathrm{B}}T}$. Because the equations are phenomenological, we wanted to minimize the number of fit parameters necessary to describe the data, and found that only two of the terms needed to be scaled by a proportionality constant to still provide good agreement with the data (correlation is shown in Fig S4).

Minor Point 7:

Referee: *The second movie would benefit from drawing the triskelia with thicker lines.*

Reply: Yes, we remade the movie S2 according to your suggestion. (Movie S2)

Minor Point 8:

Referee: *How is E defined; is is the theoretical maximum or the actual maximum achieved in the simulations?*

Reply: *E* is not a theoretical maximum. *E* is extracted from the fit to the kinetics observed in simulation, so it represents the expected maximum achieved if the simulation progresses enough long. We have clarified this on Page 20.

Minor Point 9:

Referee: *Fig 3 is referred to before Fig 2, Fig 6 before 2 through 5, etc.*

Reply: Thank you for pointing these out--the inappropriate orderings are now corrected so that all Figs are referenced in order.

Page Break

Reviewer #2:

The authors study the assembly of clathrin-coated structures on lipid membranes, using a structure-resolved reaction diffusion equation solver framework that they have developed. They particularly focus on the poorly understood experimental observation that the critical nucleus size of such clatherin-coated structures is surprisingly large, containing on the order of 20 or more clathrin subunits. Through extensive modeling, they determine factors that control the stability of clathrin-structure intermediates; in particular that excess adapter protein is crucial for enabling nucleation.

The article is for the most part thoroughly and clearly written, the modeling is comprehensively described, and the relevance of the results is strongly supported by comparisons against recent in vitro experimental measurements.

The manuscript also provides a good description of previous work, placing the current work in the proper context.

The simulation results provide a detailed understanding of how clathrin assembly depends on relevant control parameters, and the prediction that excess adapter concentrations are required for nucleation is an important observation which is unlikely could be made through experiments alone.

General Reply: We thank the reviewer for taking the time to review our manuscript, providing these positive comments, and raising the following points. Please find our itemized responses below and marked revisions highlighted in yellow in the manuscript.

I have a few minor comments on the text:

Specific Reply to each comment:

Minor Point 1:

Referee: *-for the benefit of readers with limited cell biology knowledge, the authors might include an additional sentence or two about why understanding clathrin assembly is biologically important.*

Reply: Good suggestion: we added a few sentences at the beginning of the introduction to address the biological importance of understanding clathrin assembly. (Page 4)

Minor Point 2:

Referee: -*The description of the reaction-diffusion equation solver framework could be described in a bit more detail, although it is noted that these descriptions are in prior works.*

Reply: Yes, we have now added more details about the reaction-diffusion framework by outlining the solver in two new paragraphs in Models and Methods (Pages 8), as well as adding several sentences throughout the rest of the Models and Methods to clarify the effects of orientation and dimensionality on the model implementation and reaction dynamics. (Pages 9-12).

Minor Point 3:

Referee: - *page 9, "When two molecules bind via their specific interaction sites, they adopt a pre-specified orientation relative to one another."*
Can the authors elaborate on this? Does this mean that when two interaction sites come into contact, they immediately reorient into some desired orientation, regardless of the contact angle? How does this affect association kinetics?

Reply: Yes, the reviewer is correct that when an association event occurs (and not otherwise), the two molecules reorient into the desired orientation of the bound complex regardless of contact angle. We have now added a new paragraph to elaborate on this in the paper on Page 9. The reason that the binding is independent of contact angle is to ensure that we can reproduce the expected association kinetics based on the model's reaction parameters. This was characterized in detail in our previous work, with comparison to nonspatial kinetic models (Johnson, M, *J Phys Chem B*, **122**, 11771-11783 2018).

We note that one consequence of this decision is that if two large complexes associate, then large displacements of components in the complex are possible to align the binding sites. We thus reject moves that cause unphysically large displacements for components of a complex following these association events. This rejection criteria is enforced by a cutoff value, where association that result in shifts of an interface on either complex by *scaleMaxDisplace* * <RMSD> is rejected. <RMSD> is calculated from $(6*D_{\mathrm{eff}}*\mathrm{d}t)^{0.5}$ in 3D and $(4*D_{\mathrm{eff}}*\mathrm{d}t)^{0.5}$ in 2D, where D_{eff} is the effective translational diffusion coefficient of one complex (including the contribution from rotational diffusion). We ran simulations to determine how this choice of rejection criteria would impact the kinetics, and found that the kinetics of the full assembly system were largely unchanged as long as *scaleMaxDisplace* was >~10. This data is now shown in a new supplemental Figure S2. Thus we set our value to 10 in our simulations. The main consequence of this rejection criteria is that the sizes of clathrin clusters are somewhat smaller, as most large annealing events are rejected (new Figure S2).

<u>Minor Point 4:</u>

Referee: *-Relatedly, can the authors describe more clearly the limitations of the algorithm; for example, to what extent does the model account for incorrectly oriented clathrin-clatherin interactions and how might this affect results.*

Reply: For the flat clathrin lattices (all the *in vitro* and physiologic-like simulations on membranes), the hexagonal lattices assemble free of defects, so all clathrin are oriented properly relative to their binding partners. However, for the spherical cage forming lattices in solution, the rigid clathrin trimers cannot perfectly tile the spherical surface, and thus some of the clathrin-clathrin contacts are not perfectly aligned, as the reviewer notes.

We allow these imperfect contacts to still form bonds within a cutoff distance, to mimic the structural flexibility of real biological molecules. Currently we do not have a more sophisticated treatment of the rigid assemblies to account for these defects. The choice

of the cutoff distance can have a (relatively minor) influence on the final equilibrium reached, by not stabilizing some of the contacts in the growing lattice. On page 10, we added comments on this effect: ". Imperfect contacts are still able to form bonds at a specified cutoff of 5.5nm (SI Methods), which contributes to stabilizing the lattice. In previous work we tested cutoff distances that support physically reasonable lattice structure with minor effects on assembly kinetics[47]."

Minor Point 5:

Referee: -*Can the authors describe in more detail the motivation for setting \Delta G_strain=6.9? It is not clear how the authors settled on such a precise value.*

Reply: Yes, we actually chose the values of $f = \exp\left(-\frac{\Delta G_{\mathrm{strain}}}{k_{\mathrm{B}}T}\right)$, and varied f across 1, 10^{-3} and 10^{-6}. $\Delta G_{\mathrm{strain}} = 6.9 k_{\mathrm{B}}T$ corresponds to $f = 10^{-3}$. We clarified this in the paper on Page 13.

Minor Point 6:

Referee: -*A positive aspect of the modeling is that many of the parameters are fixed by experiment. This does leave six parameters to be optimized. From the description given in the text, the optimization procedure seems rather unsystematic. Can the authors provide estimates of sensitivity of the results to optimized parameter values, or the extent to which the fit parameter values are globally optimized?*

Reply: On page 17 we have now added several sentences to provide more detail on the optimization. Including "We chose not to use learning-based or unguided global optimization approaches, as it was straightforward to run many simulations simultaneously along a multi-dimensional grid of parameters." We add that the sensitivity of the lag-time and the steepness of growth parameters can be estimated relatively well using Eqs 2 and 3: "The sensitivity of the macroscopic kinetics to each parameter can be relatively reliably estimated by the data and functions shown in Fig 3D (and Fig 4B), as quantified in Eqs. 2 and 3."

Since these Eqs apply to both the *in vitro* and physiologic-like simulations, they describe the functional dependence of the macroscopic kinetics to the parameter values in both cases.

Minor Point 7:

Referee: -*"Transport We estimate transport properties from Einstein-Stokes, with DCla=13m2/s, DR,Cla=0.03 rad2/s, Dap=25m2/s, DR,ap=0.5 rad2/s, Dlipid = 0.5m2/s, and DR,lipis=0.01rad2/s, to allow bound complexes to rotate on the surface. Diffusion slows as complexes grow, consistent with Einstein-Stokes29. For example, adaptor proteins on the membrane have a translational diffusion constant of 0.49m2/s."*
Can the authors elaborate on this? Where do these diffusion constant values come from? What does it mean that "Diffusion slows as complexes grow, consistent with Einstein-Stokes"? Is this based on hydrodynamic radius of a complex?

Reply: Yes, our definitions of diffusion constants are based on the hydrodynamic radii of the molecules, and we assume that the radii sum as the complex grows. We have now added in text on page 10 to make this more clear: "Diffusion slows as complexes grow, consistent with the assumption that the hydrodynamic radius of the complex is the sum over constituents, and using the Stokes-Einstein relations[31]. Specifically, for a complex with *N* molecules, the transport coefficients are given by: $D_{\mathrm{x}} = [\sum_{i=1}^{N} D_{xi}^{-1}]^{-1}$ and $D_{\mathrm{Rx}} = [\sum_{i=1}^{N} D_{\mathrm{R}xi}^{-1/3}]^{-3}$."

Minor Point 8:

Referee: - *p24: "For higher adaptor concentrations at equilibrium, we observe a bimodal distribution in ()… This is a notable outcome, as … after the majority of solution clathrin and adaptor are concentrated into a single coated structure, the remaining clathrin forms small clusters that are not stabilized against disassembly"*
Why is this a notable outcome? It sounds like you are just saying that at equilibrium the system undergoes macroscopic phase separation, as one would expect given that there is nothing in the model that would stop cluster free energies from monotonically decreasing with size after the initial barrier is crossed.

Reply: Yes, it is true as the reviewer notes that we are seeing a phase separation into a dense cluster and dilute remaining clathrin. We still think this is notable to emphasize, particularly as the free energy does not keep dropping monotonically with *n*, but has a 'wall' at large *n* due to not having enough adaptors to recruit clathrin to the peripheries of existing lattices. We have modified the text to make this point more clearly on page 26: "This is notable, as it demonstrates that even at higher adaptor concentration, the system does not fully transition into a single coated structure (which would be expected if the free energy monotonically decreased with increasing *n*). After the majority of solution clathrin and adaptor are concentrated into a single coated structure, the remaining and diluted clathrin forms small clusters that are not stabilized against disassembly."

Minor Point 9:

Referee: *-Fig. 5 and related text: the manuscript seems to say that the barrier to assembly ends around size n \approx 25, and is then followed by a relatively flat but noisy free energy profile, regardless of parameter values. Is this correct? Normally from classical nucleation theory the critical nucleus size would depend on binding affinity values and subunit concentrations. Can the authors explain why that is not so much the case here?*

Reply: Yes this is correct. We added more text on page 27 to speculate on why the nucleation size is independent of adaptor concentration. We note that the nucleus size does, as expected, depend on the clathrin concentration: "We note that it is somewhat surprising that the barrier to nucleation n_1 is largely independent of adaptor concentration. However, the clathrin concentration in all simulations is the same, suggesting that the barrier is largely encoded by the clathrin concentration and lattice structures formed, with the critical nucleus requiring sufficient clathrin cross-links to form and adaptors only able to bias towards assembling these structures. Indeed, with changes to clathrin concentration, the size of initially stable nuclei n_1 does change, indicating that it is the total clathrin available that controls the initial barrier to nucleation (Fig. S6)."
